# Construction of a novel shuttle vector for use in *Haemophilus influenzae* and *H. parainfluenzae*

**DOI:** 10.1016/j.mimet.2010.09.010

**Published:** 2010-12

**Authors:** Esther Robinson, Mario Juhas, Derek Hood, Derrick Crook

**Affiliations:** a Nuffield Department of Clinical Laboratory Sciences, University of Oxford, UK; b Department of Microbiology, University of Zurich, Switzerland; c Nuffield Department of Clinical Medicine, University of Oxford, UK

**Keywords:** Allelic complementation, *Haemophilus influenzae*, *Haemophilus parainfluenzae*, pEJ6, Shuttle vector

## Abstract

*Haemophilus influenzae* is an important human pathogen. A number of complete genome sequences of various haemophili are available; however, functional studies have been limited by the lack of an effective shuttle vector which functions in all strains. Here, we have constructed a shuttle vector, pEJ6, which transfers genes between *Escherichia coli* and *H. influenzae* and *H. parainfluenzae*. The vector contains an origin of replication from pLS88 which is functional in *E. coli* and *H. influenzae*. In addition it contains an RP4 mobilisation region. The vector can be introduced by electroporation and conjugation into capsulate and non-typeable *H. influenzae* and is functional for allelic replacement and mutant complementation. The vector will be useful for investigating gene function in *Haemophilus* spp.

## 1 Introduction

*Haemophilus influenzae* is an important human pathogen. The capsulated form (principally *H. influenzae* type b, or Hib) causes invasive disease including meningitis and bacteraemia with significant morbidity and mortality. Non-capsulated, or non-typeable *H. influenzae* (NTHi) causes diseases such as otitis media, which are responsible for a large burden of disease in the community. The full genome sequence is now available for a number of strains (Fleischmann et al., 1995; Hogg et al., 2007), but relating genomic information to pathogenesis remains a challenge. *Haemophilus parainfluenzae* is a closely-related species, regarded as non-pathogenic. Comparisons between *H. influenzae* and *H. parainfluenzae* are therefore expected to provide important insights into pathogenesis.

Molecular genetic investigation of *H. influenzae* and other *Haemophilus* spp. has been seriously limited by the absence of a reliable vector based genetic system. The ideal vector is small, amenable to genetic manipulation, easily introduced into a variety of clinical and laboratory strains and stable therein.

This study describes the development of a family of vectors based on the origin of replication of the *Haemophilus ducreyi* resistance plasmid pLS88 (Willson et al., 1989; Dixon et al., 1994) and the mobilisation region of the RP4 plasmid (Simon et al., 1983), present in the *Salmonella* suicide vector, pGP704 (Miller and Mekalanos, 1988). The RP4 mobilisation region allows vector transfer by conjugation from hosts containing compatible conjugation machinery, such as strain SM10 λpir (Miller and Mekalanos, 1988; Daines and Smith, 2001). This mobilisation system was previously used in an *Escherichia coli* to *H. influenzae* shuttle vector (Daines and Smith, 2001). pGP704 contains an R6K origin of replication which is dependent on the presence of a “*pir*” gene (Inuzuka and Helinski, 1978). The native plasmid R6K contains this gene, encoding a protein, π, which binds to DNA during replication initiation. pGP704 lacks the *pir* gene, so that it cannot replicate unless the host strain expresses the π protein. A number of *E. coli* strains are available with this gene. In the absence of an additional origin of replication, the vector is unable to replicate in *Haemophilus* spp.

The vectors we have developed are readily introduced into *H. influenzae* strains, including Rd, Hib and non-typeable (NTHi) as well as *H. parainfluenzae*. Vector transfer by conjugation and electroporation are possible, providing a variety of approaches to increase success. Transformation into *Haemophilus* spp by the MIV method (Poje and Redfield, 2003) is also possible, but at low efficiency, as the double stranded DNA is circular. The vectors have demonstrated utility in expression analyses, as well as for complementation and allelic replacement. This provides an important new tool set for researchers investigating gene function in *Haemophilus* spp*.*

The vector family described here is being used to investigate the function of ICE*Hin*1056 (Dimopoulou et al., 2002), an integrating and conjugating element found in *H. influenzae* b.

## 2 Materials and methods

### 2.1 Bacterial strains, plasmids and growth conditions

Bacterial strains used are listed in Table 1. Plasmids are described in the text. *Haemophilus* spp were grown on Hib medium (Columbia agar supplemented with 5 g/l yeast extract, 15 μg/ml NAD and 15 μg/ml haemin) or in brain–heart infusion (BHI) broth supplemented with 15 μg/ml NAD and 15 μg/ml haemin (sBHI). *E. coli* was grown on Luria-Bertani agar or in Luria-Bertani broth. When appropriate, media were supplemented with antibiotics as follows, for *H. influenzae* and *E. coli* respectively:

Ampicillin 4 μg/ml, 100 μg/ml; gentamicin 2 μg/ml, 5 μg/ml; kanamycin 10 μg/ml, 50 μg/ml; erythromycin 20 μg/ml, 300 μg/ml; nalidixic acid 10 μg/ml, 20 μg/ml; tetracycline 2 μg/ml, and 12 μg/ml.

Plasmid preparations were performed using the Miniprep spin© or Midiprep spin© kits as per manufacturer's instructions (Qiagen, UK). Chemicals were supplied by Sigma (Poole, UK).

### 2.2 PCR amplification

PCR amplification was carried out using the Qiagen Taq Mastermix kit for standard PCR and Qiagen PFU PCR kit (Qiagen, UK) for proofreading PCR. PCR conditions used for the amplification of our fragments were as follows: 94 °C for 3 min, then 35 cycles of 94 °C for 30 s followed by 55 °C for 30 s and 72 °C for 1 min per kb of DNA; final extension 72 °C for 10 min.

### 2.3 Restriction digests and ligations

Restriction and ligation enzymes were supplied by New England Biolabs (NEB UK) and used according to manufacturer's instructions.

### 2.4 Preparation of electrocompetent cells

*E. coli* was rendered electrocompetent using the method of Miller and Nickoloff (Miller and Nickoloff, 1995). For *H. influenzae* and *H. parainfluenzae*, the method of Bakaletz et al. (Bakaletz et al., 1988) was used.

### 2.5 Electro-transformation

This was carried out using a Bio-Rad© micropulser on setting Ec2 for *E. coli* and *Haemophilus* spp. Procedures used were as detailed in the manufacturer's manual for *E. coli*. The method was the same in *H. influenzae* and *H*. *parainfluenzae* except that the recovery medium was sBHI and the incubation time before plating was 2 h.

All experiments to measure transformation efficiency were carried out in triplicate.

### 2.6 Construction of pEJ6 shuttle vector

**Fig. 1:**
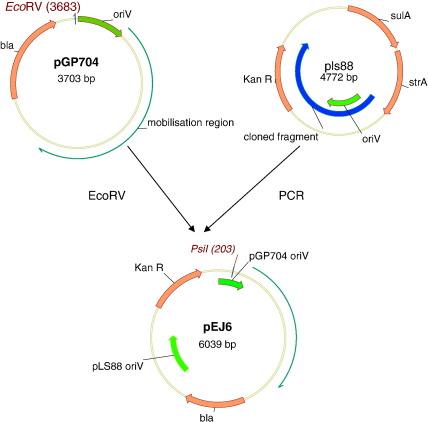
Construction of shuttle vector pEJ6.

Plasmid pGP704 was first digested with EcoRV. Subsequently, the digested plasmid was ligated with a fragment amplified from pLS88 using proofreading polymerase (see Fig. 1). The amplified fragment contained both the oriV and the kanamycin resistance gene. The primer sequences used for amplification of the fragment were as follows: forward 5′-gccttcgctgtcctattcaa and reverse 5′-accctttttgtgtccttgct. The ligated mixture was then transformed into electrocompetent *E. coli* DH5α and transformants selected on kanamycin-containing medium.

The resulting recombinant plasmid was extracted from transformants and its identity checked by restriction digestion. This plasmid was designated pEJ6 (see Fig. 1).

Vectors were designed and vector maps drawn in Vector NTI© (Invitrogen, UK).

### 2.7 pEJ6 vector derivatives

**Fig. 2:**
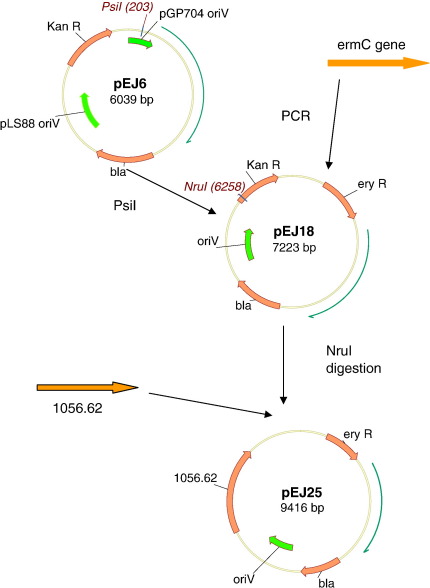
Construction of the vector family.

An ermC gene (van der Voort et al., 1996) was amplified from pER2 (forward primer: 5′-gatccccggccgtgcaggaattcgatatcaagc; reverse primer: 5′- ccgggccggccgtcgaggtcgacggtatcg) and ligated into PsiI-digested pEJ6 to generate plasmid pEJ18. Further derivatives were created for cloning experiments (Fig. 2).

### 2.8 Conjugation experiments

Conjugation experiments were carried out as previously described (Juhas et al., 2007). Experiments were carried out in duplicate.

### 2.9 Vector stability

A modification of the method used for vector stability assays in *E. coli* was performed (Daines and Smith, 2001). Experiments were carried out in duplicate.

### 2.10 Sequencing

Sequencing was performed using a capillary ABI-3730 DNA analyser (Applied Biosystems, UK) with primer sequences as follows: forward primer 5′-gggataataccgcgccacat; reverse primer 5′-ccattcaacgacgtaaacag.

### 2.11 Allelic replacement by suicide vector

A suicide vector, pEJ16, was created by cloning a mutant Hib1056 gene, 1056.62, into the EcoRV site of pGP704. 1056.62 was disrupted by insertion of a kanamycin cassette into the middle of the open reading frame in vector pGEM-TraI-Kan (Dr M Juhas, unpublished). pEJ16 was transferred by electroporation into two recipient *H. influenzae* strains carrying wild type 1056.62 genes: the wild type Hib 1056 and an Rd strain harbouring the ICE*Hin*1056 (Dimopoulou et al., 1997, 2002), ID1. In addition, pEJ16 was introduced into strain ID1 by conjugation thus creating strain RE21.

Absence of the suicide vector was confirmed by PCR using two plasmid-specific sequences in strain RE21 and the other strains created by transformation of pEJ16. Primer sequences were: forward primer 1 5′-agggcttctaaaacgccttc, reverse primer 1 5′-gatccagcagttcaacctgt, forward primer 2 5′-ggttgctggcgcctatatc, reverse primer 2 5′-gctctgatgccgcatagtta.

Allelic replacement of the wild type 1056.62 gene was confirmed using primers located within the cloned fragment ( forward primer: 5′- tacctgaaaaggccaaaagg, reverse primer: 5′-caactgctttacgccattga) and also on the chromosome of Hib1056 (forward primer: 5′- tagccgctgcctagaactccctc, reverse primer 5′-gaaacaatcggcagatgctcagc). PCR using these primers demonstrated that a double crossover event had occurred in the transformed strains leading to allelic replacement.

### 2.12 Complementation of mutation

A complementing vector, pEJ25, was constructed, containing a wild type 1056.62 gene inserted into the *Nru*I site of pEJ18 (Fig. 2). pEJ25 was electroporated into strain RE21. RE 21 harbours ICE*Hin*1056 with a kanamycin cassette disrupting 1056.62. Conjugation frequencies for the mutant strain RE21, the complemented mutant RE21 harbouring pEJ25 and RE21harbouring a control vector, pEJ18, lacking the gene 1056.62, were measured.

## 3 Results

The shuttle vector, pEJ6, was constructed by ligating a PCR-amplified fragment of pLS88 into EcoRV-digested pGP704 (Fig. 1).

pEJ6 was transferred by conjugation, electroporation or MIV transformation into *H. influenzae* Rd (Table 2). Vector transfer into haemophili other than *H. influenzae* Rd was also carried out in order to demonstrate the utility of the vector.

Transformation efficiency varied by a factor of 10^4^ depending on the source strain from which the plasmid was isolated (Table 3). However, when the same source strain was used, results were consistent between experiments. Restriction barriers would be expected to act on inter-species transfer, accounting for differences in transformation efficiency with vectors sourced from *H. influenzae* and *E. coli*. In addition, DH5α and TOP10™ have different genotypes, including differences in their restriction modification systems. TOP10 is mcrA Δ (mrr–hsd–RMS–mcrBC), DH5α hsdR17 only. In view of this and previous data on *H. influenzae* suggesting that electro-transformation is affected by restriction pattern (Mitchell et al., 1991), it is possible that restriction barriers explain the differing transformation efficiencies.

pEJ6 was isolated from recipients and its identity checked by restriction digestion. The plasmid, designated pEJ6, isolated from the initial cloning and transformation demonstrated an unexpected banding pattern after digestion with *HindIII*. Limited sequencing was therefore performed on pEJ6 and it became apparent that there had been non-specific digestion of the parent plasmid, pGP704, by the restriction enzyme EcoRV, resulting in 128 base pairs immediately 5′ of the cloning site being lost.

This vector was stable on all subsequent introductions to both *E. coli* and *Haemophilus* spp*.*. This was verified by checking restriction digest banding patterns.

### 3.1 Vector stability

Using the method of Daines et al. (Daines and Smith, 2001), plasmid pEJ6 was recoverable from over 90% of *H. influenzae* Rd after at least 100 generations in the absence of antibiotic selection, thus confirming its stability. This experiment was carried out in duplicate. Stability was not formally verified in other strains but no instability was observed during this set of experiments.

### 3.2 Allelic replacement by suicide vector

**Fig. 3:**
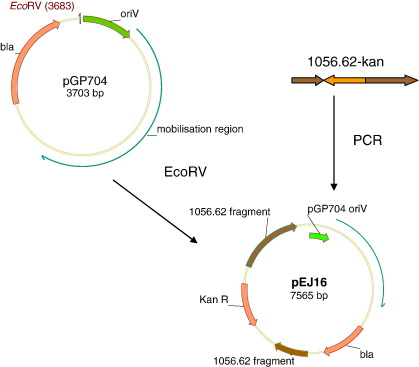
Construction of pEJ16 suicide vector.

A suicide vector, pEJ16, was constructed by ligating gene 1056.62, mutated by insertion of a kanamycin cassette into EcoRV-digested pGP704 (Fig. 3). pEJ16 was transferred into both Rd-derived and Hib1056 recipient strains. Transfer efficiency could not be determined owing to the suicide nature of the vector; only the efficiency of allelic replacement was measured. Allelic replacement occurred at frequency of 3.1 × 10^3^ mutants/μg plasmid DNA for suicide vector introduced by electroporation and 6.3 × 10^−7^ mutants/donor for conjugation in strain ID1. Electroporation into strain Hib1056 led to allelic replacement at a frequency of 1.2 × 10^1^ mutants/μg plasmid DNA.

As was found for the shuttle vector, efficiency of transfer, as measured indirectly by allelic replacement, is 100-fold less in the clinical strain, *H. influenzae* b 1056, than in *H. influenzae* Rd.

1056.62 is required for conjugal transfer of the *Haemophilus* genomic island, ICE*Hin*1056; mutation of 1056.62 conferred by allelic replacement therefore renders conjugation undetectable.

Allelic replacement was confirmed by PCR as well as measurement of conjugation frequency. pEJ16 could not be detected by PCR amplification of plasmid-specific sequences (see Materials and methods for primer sequences) or plasmid extraction by Mini-prep© from any transconjugants.

PCR amplification of the target gene, 1056.62, in the recipient strains confirmed that correct allelic replacement had occurred as the result of a double crossover event and that the gene containing the kanamycin resistance cassette was now present. PCR using Hib 1056 chromosomal primers uniformly gave a product for the transconjugants, which was of the expected size. There was no evidence on PCR analysis of a single crossover event having occurred.

### 3.3 Gene expression from the vector and allelic complementation

Antibiotic resistance genes (kanamycin and erythromycin) were stably expressed from the vector family generated in this study (Fig. 2). In addition, a green fluorescent protein cloned into pEJ6 was shown to be expressed (Woodhall et al, unpublished data).

Plasmid pEJ25 was constructed by ligating 1056.62 amplified by proofreading PCR into NruI-digested pEJ18 (see Fig. 2). pEJ25 was used to complement the mutated 1056.62 in strain RE21. 1056.62 is essential for conjugative transfer of ICE*Hin*1056 and thus functional complementation is assayed by measuring conjugation frequency to a suitable recipient. The parent strain transfers ICE*Hin*1056 at a frequency of 5 × 10^−2^ transconjugants per donor while strain RE21, in which the open reading frame of 1056.62 has been disrupted shows undetectable conjugation of ICE*Hin*1056. In the presence of the complementing vector, ICE transfer occurred at a frequency of 1 × 10^−3^ transconjugants per donor thus confirming successful complementation. The presence of a control vector (pEJ18) in strain RE21 did not facilitate conjugation, demonstrating that restoration of conjugation in the mutant strain was a specific effect of the complementing cloned gene, 1056.62.

ICE*Hin*1056 conjugates at high frequency from ID1 as previously demonstrated (Juhas et al., 2007). Mutation of 1056.62 renders conjugation frequency below the limit of detection, while complementation of the mutation with pEJ25 restores conjugal transfer of ICE*Hin*1056. Furthermore, pEJ25 was recoverable from complemented mutants demonstrating that the vector persists. PCR demonstrates that the mutated 1056.62 is still present on the chromosome, so allelic replacement is not responsible for restored conjugation frequency.

## 4 Conclusions

This study provides a first example of a shuttle vector system applicable in *Haemophilus* spp, which is functional for allelic complementation. It is notable that transformation efficiency varied greatly depending on the source of the donor plasmid. It is not surprising that *H. influenzae* to *H. influenzae* transfer is more efficient, as it would be expected that restriction modification systems are compatible. Although, in Rd, effective vector transfer was achieved whatever the source of the plasmid, in clinical and pathogenic strains, vector transfer efficiencies are approximately 100-fold less than for Rd, which may account for reported vector failures in such strains. The ease of manipulation of this vector system allows passage through *H. influenzae* Rd prior to electroporation into a clinical strain, further increasing the utility of the system.

The vector system described in this paper will facilitate functional studies based on the large amount of *Haemophilus* genome information now available. It is intended in this laboratory to provide the means to investigate transfer of the ICE*Hin*1056 (Mohd-Zain et al., 2004). Ligation of the putative origin of transfer (oriT) and transfer genes into the vector family will help elucidate the mechanism of transfer for this ICE.

## Figures and Tables

**Table 1:** Bacterial strains used in this study.

Strain	Genotype/strain details	Reference/available from
*E. coli* DH5α	F′phi80d*lac*Z delta(*lac*ZYA-*arg*F)U169 *deo*R *rec*A1 *end*A1 *hsd*R17 (rk−, m k+) *pho*A *sup*E44 lambda-*thi*-1 *gyr*A96 *rel*A1/F′ *pro*AB + *lac*IqZdeltaM15 Tn10(*tet*r)	Standard laboratory strain
*E.coli* DH5α λ pir	As above, but with λ pir	Gift of Dr M Waldor
*E. coli* TOP-10	F- *mcr*A Δ(*mrr-hsd*RMS-*mcr*BC) φ80*lac*ZΔM15 Δ*lac*X74 *rec*A1 *ara*D139 Δ(*araleu*) 7697 *gal*U *gal*K *rps*L (StrR) *end*A1 *nup*G	Invitrogen UK
*E. coli* SM10 λ pir	*thi thr leu tonA lacY supE* recA::RP4-2-TC::Mu Km (*lac pro*) *argE*(Am) *rif nalA recA*56	(Miller and Mekalanos, 1988)
*H. influenzae* Rd	Non-encapsulated standard laboratory strain	(Laboratory collection)
*H. influenzae* Nf38 nal 10	Rd, rec — rendered resistant to nalidixic acid	Gift of Dr I Dimopoulou
*H. influenzae* Rd nal 10	Rd rendered resistant to nalidixic acid	Gift of Dr I Dimopoulou
*H. influenzae* b 1056	Clinical strain resistant to ampicillin, tetracycline and chloramphenicol, resistance conferred by ICE*Hin*1056	Gift of Dr I Dimopoulou (Dimopoulou et al., 2002)
*H. influenzae* XT1	Nf38 containing ICE*Hin*1056 transferred by conjugation	Laboratory collection
*H. influenzae* Strain 11	Rd containing ICE*Hin*1056 transferred by conjugation	Laboratory collection
*H. influenzae* ID1	Rd nal containing ICE*Hin*1056 transferred by conjugation	Gift of Dr I Dimopoulou
*H. influenzae* Pitt II	Non-encapsulated clinical strain	Gift of Dr G Ehrlich
*H. influenzae* RE21	ID1 with kanamycin insertion in gene 1056.62	This study
*H. parainfluenzae* T3T1	Commensal strain	Laboratory collection

**Table 2:** Summary of vector (pEJ6) transfer efficiency.

Recipient strain	Method	Efficiency
Rd	Electroporation	2 × 10^1^–8.9 × 10^5^ cfu/μg
	Conjugation (*)	5 × 10^− 2^ recipients/donor
	MIV transformation	5 × 10^2^ cfu/μg
*H. influenzae* b (1056)	Electroporation	1.2 × 10^3^ cfu/μg
*H. influenzae* PittII	Electroporation	1.7 × 10^3^ cfu/μg
*H. parainfluenzae* T3T1	Electroporation	3.9 × 10^5^ cfu/μg

(*) conjugation efficiency is expressed in different units from transformation efficiency, as conjugation does not involve introduction of a measurable quantity of DNA into a reaction. The only measurable input is donor cells.

**Table 3:** Influence of vector source on transformation efficiency of *H. influenzae* Rd by pEJ6.

Plasmid source	Efficiency (1) cfu/μg	Efficiency (2) cfu/μg	Efficiency (3) cfu/μg	Efficiency (4) cfu/μg
Nf38 (Rd derivative)	5.2 × 10^5^	9.8 × 10^5^	3.6 × 10^5^	6.1 × 10^5^
*E. coli* TOP10	6.5 × 10^3^	5.0 × 10^3^	1 × 10^4^	1.6 × 10^4^
*E coli* DH5α	2.8 × 10^1^	2.1 × 10^1^	2.4 × 10^3^	3.5 × 10^3^
